# Online incidental statistical learning of audiovisual word sequences in adults: a registered report

**DOI:** 10.1098/rsos.171678

**Published:** 2018-02-21

**Authors:** Sengottuvel Kuppuraj, Mihaela Duta, Paul Thompson, Dorothy Bishop

**Affiliations:** Experimental Psychology, University of Oxford, Oxford, Oxfordshire, UK

**Keywords:** statistical learning, verbal working memory, incidental learning, online learning, non-adjacent dependencies, probabilistic dependencies

## Abstract

Statistical learning has been proposed as a key mechanism in language learning. Our main goal was to examine whether adults are capable of simultaneously extracting statistical dependencies in a task where stimuli include a range of structures amenable to statistical learning within a single paradigm. We devised an online statistical learning task using real word auditory–picture sequences that vary in two dimensions: (i) predictability and (ii) adjacency of dependent elements. This task was followed by an offline recall task to probe learning of each sequence type. We registered three hypotheses with specific predictions. First, adults would extract regular patterns from continuous stream (effect of grammaticality). Second, within grammatical conditions, they would show differential speeding up for each condition as a factor of statistical complexity of the condition and exposure. Third, our novel approach to measure online statistical learning would be reliable in showing individual differences in statistical learning ability. Further, we explored the relation between statistical learning and a measure of verbal short-term memory (STM). Forty-two participants were tested and retested after an interval of at least 3 days on our novel statistical learning task. We analysed the reaction time data using a novel regression discontinuity approach. Consistent with prediction, participants showed a grammaticality effect, agreeing with the predicted order of difficulty for learning different statistical structures. Furthermore, a learning index from the task showed acceptable test–retest reliability (*r* = 0.67). However, STM did not correlate with statistical learning. We discuss the findings noting the benefits of online measures in tracking the learning process.

## 1. Introduction

Human cognition depends on being able to predict what will happen next, which requires the ability to detect probabilistic relationships in an incoming sequence of stimuli. A burgeoning literature on statistical learning explores the nature and extent of this sensitivity to distributional properties in the input. A major impetus for this work has been to understand how statistical learning is implicated in language development, including mastery of grammatical dependencies [1]. Statistical learning appears to involve domain-general mechanisms that are available from early in development [2], and is implicated in developing sensitivity to distributional regularities across modalities—auditory [3], visual [4] and tactile [5]—and materials—verbal [6] and non-verbal [7]. Further, statistical learning can occur incidentally without explicit awareness of the sequential information [8], thus resembling grammatical learning (e.g. [9]), where correctness of constructions can be judged despite limited insight into underlying rules.

Research in statistical learning largely revolves around two parameters: predictability, and distance between two events in a sequence [10]. Predictability between elements, measured as transitional probability (TP), is the probability of an event *b*, given that event *a* has already occurred. It is measured as the number of times that event *ab* occurs divided by the overall frequency of *a*. Distance is measured in terms of the number of other elements intervening between *a* and *b*. For example, if *ab* occurs 50 times and *a* occurs 100 times, the TP of *a* to *b* is 0.5. Elements in a sequence are categorized as adjacent (distance = 0) when *a* and *b* are next to each other and non-adjacent when there are intervening items.

Our study focuses on triplet sequences such as *A*_1_*–S*_1_*–B*_1*,*_ *A*_2_–*S*_2_–*B*_2*,*_ *A*_2_–*S*_2_–*B*_3_ and *A*_3_*–r–B*_4_, where *r* is a random item. Sensitivity to ‘deterministic adjacent’ information is the knowledge that *S*_1_ always predicts *B*_1_ (adjacent TP = 1), and sensitivity to ‘probabilistic adjacent’ information is the knowledge that both *B*_2_ and *B*_3_ can be predicted by *S*_2_ equally often (adjacent TP = 0.5). Sensitivity to ‘deterministic non-adjacent’ information is the knowledge that *A*_3_ always predicts *B*_4_ (non-adjacent TP = 1), irrespective of the intervening item.

Adjacent level statistical learning involves computation of the TP between two co-occurring (i.e. adjacent) items (e.g. [11]). One of the major debates within statistical learning is whether ability to compute TPs can be extended to non-adjacent dependency learning. A seminal study by Gómez [12] addressed this question with infants and adults using a between-subject design. The hypothesis was that learners would detect non-adjacent dependencies, that is relation between *A_D,* in a triplet pattern such as *A–r–D* (where *r* is a variable) when the adjacent TP (i.e. *A–r*) is very low (e.g. 0.3), while the non-adjacent TP between first and the third item is high (e.g. 1). Gómez's study used an artificial grammar learning (AGL) task with meaningless syllables. There were two phases: a familiarization phase and a testing phase. In the familiarization phase, participants (both adults and infants) heard a continuous stream of syllables organized in triplet sets, such as *A–r–D, B–r–E* and *C–r–F*. Variability of *r* was manipulated by drawing *r* from a pool of 2, 6, 12 or 24 elements. In order to keep the number of strings constant across triplet sets, sets with smaller strings had to be exposed more often compared to sets with larger strings. The familiarization phase was followed by a test phase that estimated participants' learning of the statistical properties of the previously presented stream, through a series of two-alternative forced choice (2AFC) response trials, each contrasting one of the triplets presented during learning (i.e. grammatical) with a ‘foil’: a group of three items that had never appeared all together in the familiarization phase. For infants, a head-turn paradigm was used. In each trial of the test, one foil and one grammatical triplet were presented, and participants were asked to decide which group of triplets appeared more familiar, given the stream they heard. The final score that represented the individual's statistical learning ability was the number of correct responses in the test phase. Participants performed better in discriminating grammatical triplets from foils when the grammatical trials were from triplets where *r* was taken from a set of 24. Gómez concluded that drop in adjacent TPs would facilitate sensitivity to non-adjacent dependencies across infants and adults, by making them more salient. However, note that Gómez's study measured offline learning.

According to Gómez's view, in a task in which both adjacent and non-adjacent events are presented, participants should develop sensitivity to non-adjacent dependencies only after demonstrating sensitivity to adjacent dependencies (see also [13]). A strong critique of this view comes from Romberg & Saffran [14]. They used a similar offline approach and examined after each block, adults' sensitivity to adjacent probabilistic and non-adjacent deterministic dependencies on a between-subject design. They found that the adults learned non-adjacent deterministic structure earlier than adjacent probabilistic structures. Note that because they manipulated both adjacent probabilistic and non-adjacent deterministic structures within a triplet, the frequency of presentation of the tokens for non-adjacent deterministic structures were four times higher than adjacent probabilistic structures. This kind of confound is hard to control for in statistical learning studies, but complicates the interpretation of the findings.

The study we describe here is the first in a programme of research designed to investigate individual differences in statistical learning in relation to children's language learning. As noted above, research to date has established that statistical learning is an important kind of implicit learning that is likely to be involved in language acquisition, and indeed, some studies have reported associations between indices of statistical learning and language acquisition (e.g. [15]) and language impairment (e.g. [16]). There are, however, difficulties in adapting implicit online statistical learning paradigms for children, because most online tasks involve repetitive responding to visual sequences (see serial reaction time tasks [16,17]) and long sequences of meaningless material may not be engaging. Note that researchers have had some success in designing offline child-friendly tasks that were capable of capturing individual differences (e.g. [18]). More recently, West *et al*. [19] raised concerns regarding the poor reliability of some of the statistical learning tasks used with children. We attempted to address these problems when developing a new online task that included a range of dependency structures amenable to human statistical learning mechanism within a single task. The paradigm employs a guided recall post-learning that also probes the developed sensitivity to each specific statistical structure.

In what follows, we explain the rationale for modifications that the present study makes to those of Gómez and Romberg and Saffran. First, we used online as well as offline measures of learning, anticipating that the course of learning may differ for different sequence types, even if the end result was the same. The offline forced-choice measures used in most studies could miss such crucial information about the learning process. Second, most prior studies used meaningless nonsense syllables. These materials have many advantages because they allow tight control of experimental stimuli, minimizing the impact of prior linguistic knowledge. However, the use of familiar words has the advantage that it reduces the need to learn novel words as well as sequential patterns between words, and so should make a statistical learning task easier, as well as providing more intrinsic interest than meaningless material. This also makes it easier to design an online task, using participants’ responses to familiar stimuli. In addition, if the aim is to model statistical learning of grammar, it could be argued that real word stimuli are more relevant than non-words, because children first learn words and then learn how they co-occur in sequences.

A third difference is that Gómez and Saffran and Romberg used between-subjects designs. While such a design is an important first step in clarifying influences on statistical learning, it is constrained and raises the possibility that participants will adopt artificial strategies, especially if they are explicitly instructed to identify patterns in the stimuli. If the learning mechanisms uncovered in these studies are to provide a realistic explanation for grammatical learning, they should operate in situations where different kinds of statistical dependencies are encountered within the same stimulus stream, so that participants cannot easily use rational strategies to work out the structure. Accordingly, we adopt a within-subjects design.

Fourth, in these previous studies, the frequency of occurrence of triplets was confounded with statistical structure. Note that in the Gómez's study, the triplet sets that had middle items drawn from smaller pool of ‘*r*’s were exposed more often than triplet sets that had middle items drawn from larger pool of ‘*r*’s. In the Romberg and Saffran study, the frequency for non-adjacent items was very high compared to adjacent probabilistic. This could have facilitated learning of non-adjacent deterministic relations. In order to estimate relative difficulty in learnability across structures, it is necessary to control as far as possible the frequency of exposure of each structure. In practice, it is impossible to equate the frequency of both predictors and targets of triplets across conditions, as learning of a probabilistic structure (e.g. *A*_2_−*S*_2_ predicts either *B*_1_ or *B*_2_) would require the same predictors to be presented twice, whereas a deterministic sequence such as *A*_1_*–S*_1_*–B*_1_ can be learned on the basis of repeated presentation of the same one triplet. The experimenter must, therefore, decide how to control the frequency of exposure of each structure, given that we expect frequency to play a role in learning [21–23]. The present study ensures that for deterministic and probabilistic conditions (i.e. ‘grammatical’ triplets), the predictor's tokens are in the ratio of 1 : 1 and target's tokens are in the ratio of 2 : 1. This allows a ‘dense’ presentation of deterministic conditions facilitating association formation in a relatively brief timeframe. In addition, we included random, non-grammatical sequences as a control. This allows us to track the impact of the different sequence types in the same person over time.

Fifth, after a period of learning, we presented two blocks where the sequence was broken, and the first two items of a triplet no longer predicted the target as before, before reverting to the earlier sequence in a final two blocks. This approach, which copies a common method in online studies of implicit learning [e.g. [14]), means that in addition to considering faster RTs as an indication of learning, we can look at rebound of reaction time (RT) when the expected target is no longer valid. This way we can distinguish general speeding up from specific sequence learning.

### 1.1. Online versus offline

Offline measures may be problematic for three reasons. First, they may confound effects of encoding efficiency and memory constraints with statistical learning. Thus, poor performance at test phase of an offline task may simply reflect weak ability to remember individual stimuli. However, it should be noted that some offline studies have used no-learning control groups to factor out potential effects of such learning on test performance (e.g. [5]). While others show that ability to remember individual items during familiarization phase has little effect on test scores (e.g. [24]). Second, to quantify learning, offline tasks use the two-alternative forced choice method, where it is necessary to repeatedly present test items and foils. This can cause interference, thereby blurring the methodological separation between intended learning during familiarization and unintended learning during the test phase. Attempts to avoid this learning/interference during the test phase have involved introducing several tests with re-familiarization phases (e.g. [25]). Third, offline tasks do not track the discovery of regularities from the stream while it unfolds, and so do not capture the learning trajectory which online measures are capable of demonstrating (see [20]). This may prove crucial in studies that focus on the learning course of two or more conditions of interest, as in the present study. For a comprehensive review, see [20]. Misyak *et al*. [26] integrated the benefits of an online prediction task with the flexibility of an offline AGL task with an AGL-SRT (serial reaction time). On this task, rather than asking participants to passively listen to a stream of words which were presented at a fixed rate, they were asked to advance the stream of words by themselves, at their own pace, by clicking upon the rectangle with the correct (target) word as soon as they heard it, with an emphasis on both speed and accuracy. They found that learning of TPs between dependencies in the triplets resulted in shorter RTs for predicted words (the last item of the triplet) relative to unpredicted words (the first or second items of each triplet). Vuong *et al*. [27] used this online procedure to familiarize their adult participants with triplets that had probabilistic adjacent and probabilistic non-adjacent dependencies (in a within-subjects design). Their task also had ungrammatical blocks with triplets that did not have regularities between the first and the last items. Their predicted ‘grammaticality effect’ was the RT difference between ungrammatical and pooled grammatical blocks. Further, they predicted that the participants would show faster RTs for adjacent probabilistic compared to non-adjacent probabilistic triplets, on the grounds that adjacent dependencies are easier to learn. They found the predicted grammaticality effect but not the predicted effect for adjacency. However, offline tests after training did reveal a performance difference between the adjacencies (adjacent better than non-adjacent) (see also [12,13]). One of the ways Vuong *et al.*'s findings could be furthered is by tracking the difference between conditions as a factor of exposure (i.e. increment in block) rather than looking at an overall RT difference between adjacent and non-adjacent conditions.

### 1.2. The present study

The present study furthers the understanding of statistical learning by presenting auditory triplets of familiar words in three types of sequences: adjacent deterministic, adjacent probabilistic and non-adjacent deterministic, while keeping the token frequency of each structure's predictors constant in a within-subjects design. Note, even though it is not unusual for past studies in sequence learning (SL) to have manipulated TPs adjacently (e.g. [23]), non-adjacently (e.g. [15]), as well as simultaneously (e.g. [21]), the present study manipulates TPs with equated predictor's frequency across structures simultaneously, and tracks learning of these structures while it unfolds. We use a target detection within serial search task procedure (see Material and methods) to examine the online implicit perceptual learning of audiovisual sequences. This method has an advantage over conventional sequence learning tasks, as it measures sequence learning independent of motor pattern learning (e.g. [28]). Further, unlike conventional learning tasks that could be long and unengaging for participants (see [29] for similar concerns), the present task includes a reward aspect to keep them motivated. The main task is followed with a surprise ‘guided’ triplet completion task, which unlike previous free recall tasks is devised to reduce memory constraints while eliciting the knowledge of the sequence [30].

In sum, we will use group data to examine relative rates of learning for three types of sequence: adjacent deterministic, adjacent probabilistic and non-adjacent deterministic, using responses to random sequences as a baseline. The adjacent deterministic condition may be regarded as a positive control, i.e. if RTs to these sequences do not decrease, and then rebound when the sequence is broken, this would mean that our task is not suitable for demonstrating statistical learning. Our pilot data suggest that this effect should be strong at the group level. The question of interest is whether equivalent levels of learning will be seen for the other two dependency types. The work of Gómez and Vuong *et al.* (offline findings) suggest that adjacent probabilistic sequences should be learned more readily than non-adjacent dependences, when both types occur within the same task. This leads us to predict that the order of difficulty of learning, from easiest to hardest, will be (i) adjacent deterministic, (ii) adjacent probabilistic and (iii) deterministic non-adjacent. This, however, is discrepant with the findings of Romberg & Saffran [14], who found non-adjacent dependencies were easier than adjacent probabilistic sequences. Yet other studies would appear to predict that adults may learn all the structures at the same rate (e.g. [13,20]). It is, therefore, by no means a foregone conclusion whether our predicted order of difficulty will be seen. We will test this predicted order using an online measure of learning—decrease in RT over blocks and will also look for a rebound in RT when the sequence is broken after presentation of six blocks so that targets are no longer predictable. We will also use an offline target prediction test with similar predictions, and ask participants if they have explicit knowledge of the sequences.

### 1.3. Individual differences

As well as analysing group data to further our understanding of factors affecting statistical learning, we use correlational analyses to establish the reliability and validity of our task as a measure of statistical learning in individuals.

Our pilot data showed learning on our task at the group level, but substantial variation from person to person, with some appearing to learn nothing, and others learning very effectively. This suggests that the task may be promising as a measure of individual differences, but only if these differences prove to be robust when participants are retested on analogous sequences using different items. We will be able to assess reliability for the same sequence type over time, as well as considering correlations in learning indices across the different sequence types. Finally, we will consider whether an individual's statistical learning ability is related to short-term memory (STM) capacity, given that memory constraints have been proposed as a factor influencing statistical learning (see [31] for review). Although the sequences that we use here contain only three items and should be well within the memory span of typical adults, in a taxing task that involves transferring information from short-term to long-term memory, we might expect some relationship between memory span and statistical learning. If this is found, then it would provide evidence that the measure tapped into a skill that extended beyond this specific task.

## 2. Material and methods

### 2.1. Determination of sample size

A sample size of 40 participants was originally determined with two considerations in mind, taking into account pilot data from nine participants (see OSF link osf.io/342rz). First, for group-level analyses, we wanted the standard error of estimates of the effect of sequence type to be small enough to be able to detect meaningful differences between the three conditions. Second, for individual differences analysis, we aim for the standard error of estimate of test–retest reliability (*r*) of a measure of statistical learning to be small enough to be confident whether or the confidence interval for the correlation fell within 0.65, which we set as a cut-off for acceptable test–retest reliability. This is in line with test–retest reliabilities observed by West *et al*. [19] for psychometric tests, which ranged from 0.57 to 0.96, and considerably higher than the test–retest reliability observed by these same researchers on SRT tasks, where values for indices of statistical learning ranged from −0.001 to 0.21 (see also [32]). We finally included 42 participants as, after in-principle acceptance, we realized we would need a number divisible by three, so the three counterbalanced routines (see below) could be given to equal number of participants.

To take first the group-level analysis, in pilot data, the RT for the different sequence types during the learning phase was substantial, given that all three effects (mean RT during random condition compared with grammatical sequence) were estimated as greater than 140 ms, with standard errors of 25–30 ms. Thus, our sample size will be more than adequate to detect an overall learning effect. To have sensitivity to reliably detect differences between the three conditions, we aimed for a standard error no greater than 30 ms for each of the three conditions during the learning phase. The simulate function from the *lmer* package in R was used to generate data using the parameters from a linear mixed model based on pilot data and this established that with 40 participants, the standard errors for the three sequence-learning estimates were approximately 29 ms.

The standard error of a Pearson correlation coefficient is a simple function of sample size. After applying the Fisher transformation to normalize the correlation coefficient, the standard error is equivalent to 1/sqrt (*N*−3). With a sample of 40 participants, we would be able to determine that a modest-sized correlation of 0.4 (CI from 0.10 to 0.63) was not within our pre-determined confidence interval band. Although estimation of the correlation is still far from precise with this sample size, we judged this would be sufficient to determine whether the measure was promising enough to take into further studies.

#### 2.1.1. Participants and testing schedule

We aimed to recruit 40 typical adults (age range: 18–40 years) who were proficient English speakers. University of Oxford Medical Sciences Division, Research Ethics Committee approved the study (Ref: R50705/RE002) and all the participants signed an informed consent, and were paid £10 for their time. Exclusionary criteria were: no self-reported history of neurological disease, learning disorder or intake of alcohol or drugs in the previous 8 h. On a first session, the verbal STM task and one of the routines (see below) of the SL task were administered. On the second session, at least 3 days later, the SL task was administered using a new set of target items, and the recall task was administered. The recall task was not given in session 1 because we do not wish participants to be cued in to the fact that there are regular sequences.

### 2.2. Materials

#### 2.2.1. Apparatus and set-up

The online statistical learning and recall tasks were programmed using Matlab version 9.1.0.441655 (R2016b) and will be run on a Microsoft Surface Pro 4 tablet computer with a 12.3′′ (2736 × 1824) touch-screen display. Participants were tested in a quiet room with the tablet placed flat on a table in front of them at a viewing distance of approximately 30 cm. Participants were told to rest their preferred palm on the edge of the tablet and hold the dedicated stylus at the centre of 2 × 2 presentation grids (see presentation) and touch the named image as quickly as they can.

##### 2.2.1.1. Verbal short-term memory

The forward digit span subtest from Wechsler Adult Intelligence Scale [33] was administered as a measure of verbal STM. In this subtest, participants were asked to repeat pre-recorded number strings of increasing lengths from 2 to 8 in the order in which they were heard. Each accurately repeated string was given a score of 1 (maximum of 16) and participants' raw scores were used for the analysis.

##### 2.2.1.2. Statistical learning task

*Overview*. The design of the task was developed on the basis of extensive pilot testing, where we experimented with different number of sequences of different types, and different task formats. Because our ultimate goal was to use the task with children, we aimed for a task that took no longer than 30 min to administer, but which would show learning in at least some participants/conditions. This led us to a task in which there was repeated presentation of sets of eight triplets, with the order of the triplets pseudo-randomized on each presentation. The participant listened as the first two members of a triplet were presented singly and named, then the third, target member was shown in a square array with three distractors and the task was to select the named item. Thus, for grammatical triplets, learning was indexed by speeding up of the response to the target, as it could be anticipated from the two prior stimuli.

The eight triplets included: two presentations of the same adjacent deterministic sequence: *A*_1_*–S*_1_*–B*_1_, one presentation of each version of an adjacent probabilistic sequence: *C*_1_*–S*_2_*–D*_1_ and *C*_1_*–S*_2_*–D*_2_, two presentations of a non-adjacent deterministic sequence: *E*_1_*–R–F*_1_, and two presentations of random sequences, each composed of three items selected from a large pool of words. More details of triplet construction are given below.

Presentation consisted of six blocks of five triplet sequence sets (with position of triplets pseudo-randomized within each set), followed by two blocks where the final, target item of the grammatical sequences was swapped with a distractor, breaking the anticipated sequence, and two further blocks where the sequence was restored.

*Materials*. Sixty-one common nouns were selected for which high-quality images were made available to us by C Hulme and M Snowling (Artist copyright—D Chesher). The nouns are mono-syllabic and had frequency (*f*) of occurrence (*f*/10 00 000 utterances) between 20 and 1000. An audio file for nouns was created from an online text to speech forum [34] (settings: language—British English, speaker—Emma, speed—medium–slow). Any empty edges from the downloaded files were removed and a native English speaker scrutinized the pronunciation in the audio files and approved of its clarity.

**Figure 1.**
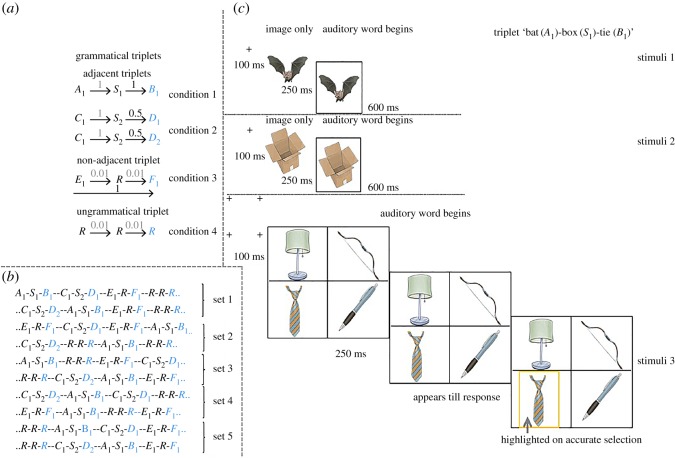
(*a*) Design of test triplets. The statistical information is monitored at the third item (in grey/blue). Shorter arrows show the forward TPs between two adjacent items. Longer arrows in non-adjacent triplet show the TPs between first and third items. On top of the arrows in bold are TPs that are of interest. (*b*) An example of triplets' arrangement within a set. Note that within set deterministic triplets occur twice and two random triplets occur. Five sets make a block. (*c*) (Print friendly) example of stimuli presentation of a triplet ‘bat(*A*_2_)–car(*S*_1_)–tie(*B*_2_)’. On the first and second stimuli, a prime appears for 100 ms followed by the image for 250 ms, followed by the auditory form of the image for approximately 600 ms, during which the image is highlighted. On the third stimuli presentation four primes appear for 100 ms, which was then replaced by four images for 250 ms. The target item is then named and pictures appear until the named item is selected. An electronic .gif motion illustration of two consecutive triplet presentations can be accessed at https://osf.io/x4td6/.

The selected 61 nouns (letters and subscripts are used for simplicity) were split into pool 1 with 7 items (*A*_1_*, B*_1_*, C*_1_*, D*_1*–*2_*, E*_1_*, F*_1_), pool 2 with 2 items (*S*_1*–*2_) and pool 3 with 52 items (*R*_1*–*52_) items. There were three possible routines, with the nouns being assigned so as to counterbalance specific items across the three grammatical conditions. Each item (i.e. noun) had an auditory and a picture form. Items 1 and 2 of the triplet are predictor items and item 3 is the target item of the triplet. The first and third items of the grammatical test triplets were formed using items from pool 1. Items *S*_1_ and *S*_2_ were used for the middle triplet item for the adjacent deterministic and adjacent probabilistic items, respectively. Note that the TPs between first and second items on adjacent triplets are always one; what is altered are the TPs between second and third items, that is, *B*_1_ has adjacent deterministic information with relevance to *S*_1_*.* Similarly*, D*_1_ and *D*_2_ have adjacent probabilistic information with relevance to *S*_2_. The non-adjacent triplet had the middle item (i.e. *R*) randomly selected from pool 3 on each presentation, therefore, dropping the TPs between second and third items to extremely low (i.e. approx. 0.01, note pool 3 has 52 items) (encouraging non-adjacent learning, see Introduction). Between the first and third items of the non-adjacent triplets, *F*_1_ has non-adjacent TP of 1 with relevance to *E*_1_. On the ungrammatical triplets (also called ‘control’ condition), all the three items were selected randomly from pool 3, thus forming very low TPs between its elements (figure 1*a*).

In summary, the design has four triplet types (henceforth, conditions). The statistical properties of interest are at the third item of a triplet, that is, the target item. The major criterion for demonstration of learning is for the RTs of grammatical items to be faster than ungrammatical items.

*Presentation*. Eight test triplets (condition 1, adjacent deterministic: *A*_1_*–S*_1_*–B*_1_*,* condition 2, adjacent probabilistic: *C*_1_*–S*_2_*–D*_1_*, C*_1_*–S*_2_*–D*_2_*,* condition 3, non-adjacent deterministic: *E*_1_*–R–F*_1_*,* condition 4, random: *R–R–R*) composed a set. Note the deterministic triplets were presented twice to match the predictor's frequency with probabilistic condition (see Introduction), and there were also two different random sequences in each set. The order of the triplets within a set was pseudo-randomized with the constraint that successive repetition of triplets from the same condition was not allowed. The task consisted of 10 blocks of 5 sets (figure 1*b*). Therefore, overall 400 triplets (i.e. 8 triplets/set × 5 sets/block × 10 blocks) are presented. Since participants give one response per triplet (see below), the task gives 400 RTs. At the seventh and eighth block, the grammatical triplet sequences are broken by designating one of the foils as a target, and the original sequence was reinstated at the ninth block. This allows us to observe an RT rebound and drop—a classical sign of learning in SRT paradigms. This design ensures that at the end of the task, each grammatical triplet is exposed 80 times (i.e. excluding blocks 7 and 8).

Trials of the learning task are presented in a paradigm that is a combination of target detection and serial search. Within each triplet, there are three stimulus presentations. All stimuli were preceded by a prime (+) for 100 ms. On the first stimulus presentation, the image (square size of 192 by 192 pixels) (i.e. first item) appeared for 250 ms followed by its auditory form. Note the auditory words had slightly varying lengths. Nevertheless, the image appeared for 870 ms (i.e. 250 ms + 620 ms of length of longest word) to ensure constant presentation duration. While the auditory word was playing, a yellow frame highlighted the image to catch the participant's attention. The second stimulus followed after the first stimulus in the same way. Note there are no distractors and the participants are not required to respond to these first two stimuli. On the third stimulus presentation (the target stimulus), four crosses are presented on 2 × 2 grid for 100 ms, which was then replaced by four images for 250 ms. The target item is then named (not highlighted) and the program waits for a response (figure 1*c*). The RT window was set to record RTs from the start of the image presentation to allow capturing any predictive responses. Therefore, any negative RTs are predictive responses (maximum limit is negative 250 ms). When responses are predictive, the audio still played the whole word. Participants are told to respond to the third stimulus by selecting the named picture as accurately and fast as possible. After an accurate response, the target image was surrounded with a yellow frame highlight signalling the end of the trial. The task is self-paced. Response accuracy is recorded, so that inaccurate RTs can be eliminated during analysis (see below). The position of the third stimulus was random for each presentation. Certain rules were applied to the third stimulus and its distractors to minimize extraneous effects on observed learning. First, they were selected in a pseudo-random fashion, with the condition that on the trials where *D*_1_ was the named picture (i.e. in the adjacent probabilistic condition), *D*_2_ was not one of the distractors and vice versa. Second, distractors did not start with the same phoneme as the target. Third, distractors were not visually similar (i.e. if ‘witch’ is the target, ‘thief’ was not one of the distractors). Fourth, the items occurring in the first two elements of the triplet cannot be one of the distractors. Fifth, out of three distractors, a minimum of two were potential targets, to ensure that the identity of the target could not be determined just by its frequency of occurrence. Sixth, the frequency of occurrence of all the third items of grammatical triplets were moderate (between 95 and 400, as per [35]) to minimize item-driven RTs.

Despite these controls, the possibility remains that some sequences will be intrinsically easier to learn than others. To control for that possibility, we devised three different routines so that the items included in triplets were counterbalanced between subjects. For instance, if *bat–car–tie* was the adjacent deterministic triplet in routine 1, it would be one of the adjacent probabilistic triplets in routine 2, and *bat-r-tie* would be the non-adjacent deterministic triplet in routine 3. Three different routines were formed using entirely different item combinations for use in session 2 (see https://osf.io/ht3wg/ for all 6 routines).

The top quarter of the screen was used to display earned visual rewards for faster responses to target stimuli. Twenty practice items with random triplet sequences were included to familiarize the participants with all the image–name pairs and the procedure of searching for and responding to the target. Participants were not informed that stimuli occur in triplets, but an inter-triplet pause of 200 ms is added to make sequences more salient (pilot testing indicated that sequences were too difficult to detect without this modification). Participants were encouraged not to stop during the blocks, but were allowed short breaks (maximum approx. 120 s) between blocks if needed. The median of the 20 practice RTs is used as a starting reference RT and a rolling median for every set is taken as reference RT from there on. Faster responses on test trials are rewarded with reference to the median RT of the previous reference RT. That is, a response was rewarded, if it is at least 10 ms faster than the reference RT. The reward criterion is, therefore, adaptive to the participant's natural speed. The reward section of the screen started as a clean slate for every block, but with a header, ‘*your score*’: which is updated once after completion of every block*.* The final number of rewards the participant earns is given at the end of the task. Immediately after the search task, participants were checked for their awareness to the repeating test triplets (by asking: ‘*Did you notice any repeating patterns?’)* and responses were scored as ‘yes’ (=1) or ‘no’ (=0) for each question. This section has no predictions hence will be reported qualitatively.

*Post-test recall task*. A guided recall task was given after the learning trials of the second test session, without forewarning. Triplets were presented as in the learning task, except that the spoken name of the third stimulus was omitted, and an additional six random items were presented as distractors to reduce the chance of correct performance by chance. The nine pictures were in a 3 × 3 grid. Participants were asked to take their time and respond by selecting the picture that they think most likely to follow the previous two words, given the stream from their previous task. If they do not know or were not confident of the target, they were asked to select the one that comes to their mind first. The probability of getting one item correct response by chance is 0.11 (i.e. one target among nine items). To ensure that difficulty of probabilistic and deterministic items is equated, deterministic items were presented twice, with a pass (score = 1) being given only if responses to both the presentations of a particular type were accurate, which would happen only for 1% of cases (*p* = 0.11 × 0.11) if responding was random.

##### 2.2.1.3. Preparing the reaction time data

*Outliers*. From the online task, inaccurate RTs were removed and RTs greater than 2000 ms were recoded to 2000 ms. This cut-off, which was selected on the basis of inspection of pilot data, excluded less than 5% of total RTs. However, note that we would adopt a different cut-off in future studies with children to reflect their overall slower RTs. From the two triplets in each condition and set, we take the one with the smallest RT—pilot testing indicated this procedure was effective in reducing noise in the data. None of the data points will be eliminated from the recall task.

##### 2.2.1.4. Statistical analysis

For analysis of group data, we specified a linear mixed effects model that includes fixed effects for phase (learning and break-sequence), triplet type (random, adjacent deterministic, adjacent probabilistic and non-adjacent deterministic), slope across the break-sequence and the interaction between them. The baseline condition for triplet type is the random condition, and the baseline condition for phase is the break-sequence phase. Thus, the interaction term indexes the mean difference in RT across triplet types and phases. We use a regression discontinuity model structure that is more commonly used in epidemiological contexts or to assess the impact of clinical or educational interventions when experiments are not feasible [36,37]. The basic idea is that data can be divided at a cut-off point at which a change has occurred, leading to the expectation that the regression of score on time will change. With this approach, we can model only two phases of the data, so we have reduced the analysis to focus on the learning and break-sequence phases. Here, there is a clear expectation that during the learning phase, we should see a decrease in RT, with a rebound when the sequence is broken at the break-sequence phase (theoretically, an extension to the model to incorporate three phases and two discontinuities is possible but beyond the scope of this study as it would require further methodological development). We have specified a linear fit, though we also explored the possibility of using a non-parametric approach which gives a closer fit to obtained learning trajectories. However, the amount of data in the pilot phase was not sufficient to explore this fully.

The slope of learning across sets was treated as a random effect. There were 50 sets in total (5 sets for each of 10 blocks). Because of the two-phase design (regression discontinuity), we re-specified the set index variable into two variables, b1 and b2, for phase 1 (learning phase) and phase 2 (break phase), respectively. This allows for the correct estimation of regression line for each phase under one estimation process. More specifically, set index was centred at zero, so that the point of discontinuity is zero. That is, b1 had negative indexes until the discontinuity for the learning phase, but then fixed zero values after the discontinuity in the break-sequence phase. For the break-sequence phase, b2 variable was coded as zero prior to discontinuity and positive set values in the break-sequence phase, as is typical in regression discontinuity design. Our extension to this model is to allow random effects for individual slopes rather than estimation of only a specific ‘treatment’ effect. These variables entered into the analysis together with the interaction with triplet type; so that we could estimate different random slopes by triplet type for each participant (see osf.io/342rz for analysis script). The analysis was run in R using the lme4 package [38].

The data from the offline recall task are very sparse, consisting of just one data point per condition. The probability of being correct by chance on each sequence type is 0.11. The data for each participant will be coded on the three conditions as pass/fail, leading to eight possible patterns of performance (1,1,1; 1,1,0; 1,0,1; 1,0,0; 0,1,1; 0,1,0; 0,0,1 and 0,0,0). The first and last patterns are uninformative about the order of difficulty of sequence learning, but we can divide the remaining six patterns into two that are consistent with the order of difficulty we have predicted (1,1,0 and 1,0,0) and four that are inconsistent (the remaining patterns). If our posited order of difficulty is correct, we predict that the proportion of participants who show patterns 1,1,0 or 1,0,0 will be greater than 0.33, which is the proportion expected if all patterns are equally likely to occur. We will test this prediction using a *χ*^2^ test, with one degree of freedom. Assuming that we have useable data from 30 participants (i.e. excluding those with patterns 1,1,1, or 0,0,0), we would be powered to detect a departure from expected probabilities if 50% or more of participants were coded as 1,1,0 or 1,0,0.

For the individual differences analyses, we need a measure that reflects learning in each participant. Although we could in principle use the estimated random effect slopes from the multi-level model for this purpose, we wanted a measure that could potentially be derived for individuals in a future clinical context. Pilot testing suggests that a regression discontinuity approach will be optimal for this purpose, as this allows us to assess the difference in slope between the training phase and the break-sequence phase for an individual participant's data. Using pilot data, we found that for participants and conditions where learning was evident by visual inspection, the test for difference in slopes from the R package RDDtools [35] gave clear results, with *t*-values greater than 2.0, whereas for those participants and conditions where there was little or no evidence of learning, much lower values were obtained. We, therefore, plan to use the *t*-value from this analysis as the index of learning in the test–retest reliability analysis, and also when computing the correlation between learning and verbal STM. Further, in response to a reviewer suggestion, we will also explore the validity of our RDD approach in testing test–retest reliability by comparing it against reliability calculated from a more traditional learning index, mean log RT difference between grammatical and ungrammatical condition.

### 2.3. Specific hypotheses and corresponding outcomes

#### 2.3.1. Do participants show a grammaticality effect in the learning task?

*Hypothesis: A grammaticality effect is anticipated with larger effects in grammatical conditions compared to the ungrammatical condition*.

The main effects of grammaticality conditions are fixed effects and can be extracted from the linear mixed effects model output. These can be interpreted as the average difference in RT between the baseline condition (i.e. random) and each grammaticality condition. If the corresponding confidence intervals do not include zero and are suitably narrow, this would confirm our hypothesis.

#### 2.3.2. What is the order of difficulty of learning?

*Hypothesis: The anticipated order of difficulty of learning, from easiest to hardest, will be adjacent deterministic, adjacent probabilistic, non-adjacent deterministic*.

For this analysis, we extract parameters from the linear mixed effects model to determine the anticipated order of difficulty of learning. Specifically, we are interested in the three interactions between grammaticality condition and phase (with the random condition and sequence-break phase acting as reference conditions). The magnitude of effect size for each interaction should differ for each grammaticality condition, the order of magnitudes will determine the difficulty of learning, with differences in learning being meaningful if the 95% confidence intervals around the estimates do not overlap zero.

#### 2.3.3. Are effects of adjacency and probability reflected in performance on the offline task?

*Hypothesis: The proportion of participants scoring ‘pass’ will be higher for deterministic than for probabilistic sequences, and higher for adjacent than non-adjacent sequences*.

As described above, the pattern of performance on the three grammatical sequences will be coded for each participant, and a *χ*^2^ test is used to determine whether patterns consistent with prediction occur more often than would be expected if all patterns were equally likely.

#### 2.3.4. Does variation in sequential learning reflect stable individual differences, or is it more determined by uncontrolled task factors?

*Hypothesis: A global measure of sequential learning will be a stable measure of individual differences*.

To assess stability of sequential learning, we will extract an overall index of learning from all the grammatical sequences, using the regression discontinuity approach described above, for each of the two test sessions for each participant, and then compute the correlation between these. A correlation coefficient of 0.65 or more will be taken as evidence of adequate test–retest reliability.

#### 2.3.5. Is statistical learning capacity affected by STM?

*Hypothesis: A positive correlation will be observed between rate of learning and verbal STM in participants*.

We predict a positive correlation between the learning rate for the three grammatical conditions and verbal STM. We are not able to give any estimate of the size of correlation to be expected, so this part of our analysis may be regarded as exploratory.

## 3. Results

### 3.1. Registered—confirmatory

We report the analysis for all 42 participants. Analyses based on the original sample size of *n* = 40 do not alter the conclusions (see model outputs and *χ*^2^ results on *n* = 40 at https://osf.io/zcdva/).

### 3.2. Do participants show a grammaticality effect in the learning task?

**Figure 2.**
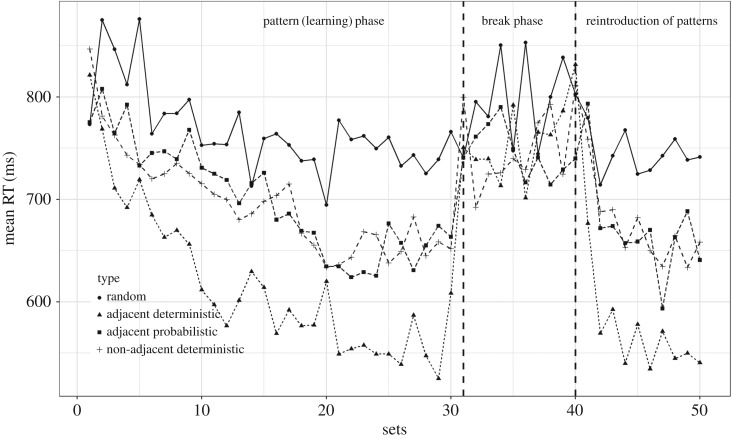
Summary plot showing learning across conditions (session 1).

In this section, we report the learning data from session 1. Grammaticality type (type 1 = reference/random/ungrammatical, type 2 = adjacent deterministic, type 3 = adjacent probabilistic, type 4 = non-adjacent deterministic), slope of learning phase, slope of break phase, grammaticality type × slope of learning phase, and grammaticality type × slope of break phase were the fixed effects. By-subject and by-grammaticality type × subject were the random effects in the model. Table 1 shows the output of the mixed effects model. A negative *β* term corresponds to a reduction in RT. The main effects are not of interest as they include both pattern and break phases: our key hypotheses concern the interaction between grammaticality type and learning phase, which are shown in bold. The *t*-values for these confirm that the adjacent deterministic and adjacent probabilistic types were learned significantly compared to the reference type (table 1, session 1 and figure 2), whereas the non-adjacent deterministic condition did not differ from the random condition.

**Table 1.** Output of the regression model for sessions 1 and 2. Registered comparisons are given in bold.

	session 1			session 2		
	estimated coefficients (*β*) ± s.e.	95% CI (*β* − 1.96 s.e., *β* + 1.96 s.e.)	*t*	estimated coefficients (*β*) ± s.e.	95% CI (*β* − 1.96 s.e., *β*+1.96 s.e.)	*t*
fixed effects						
intercept	770.5 ± 16.62	738.5, 802.5	46.36***	719.1 ± 16.22	687.3, 750.9	44.32***
adjacent deterministic	−53.8 ± 23.50	−99.8, −7.7	−2.29*	19.5 ± 22.94	−25.5, 64.5	0.85**
adjacent probabilistic	−1.4 ± 23.50	−47.5, 44.6	−0.06	−15.1 ± 22.94	−60.1, 29.9	−0.66
non-adjacent deterministic	−45.8 ± 23.50	−91.9, 0.3	−1.95	−13.3 ± 22.94	31.6, −58.3	−0.58
learning phase	−57.8 ± 18.39	−93.8, −21.7	−3.14**	−56.7 ± 17.90	−91.8, −21.6	−3.17**
break phase	4.5 ± 2.68	−0.7, 9.8	1.69	−0.2 ± 2.61	−5.3, 5.0	−0.06
**adjacent deterministic** × **learning phase**	−**132.5 ± 25.79**	−**183.1, −82.0**	−**5**.14***	−280.3 ± 25.06	−329.4, −231.1	−11.18***
**adjacent probabilistic** × **learning phase**	−**88.2 ± 25.79**	−**138.8, −37.7**	−**3**.42***	−134.6 ± 25.06	−183.8, −85.51,	−5.37***
**non-adjacent deterministic** × **learning phase**	−**42.4 ± 25.79**	−**92.9, 8.2**	−**1**.**64**	−157.2 ± 25.06	−206.3, −108.1	−6.27***
adjacent deterministic × break phase	3.0 ± 3.70	−4.4, 10.5	0.80	−3.6 ± 3.70	−10.9, 3.6	−0.98
adjacent probabilistic × break phase	−8.8 ± 3.70	−16.2, −1.4	−2.32*	0.7 ± 3.70	−6.6, 7.9	0.19
non-adjacent deterministic × break phase	0.3 ± 3.70	−7.1, 7.8	0.09	4.1 ± 3.70	−3.2, 11.4	1.11
random effects				random effects		
subject: variance = 15.31, s.d. = 3.91				subject: variance = 12.01, s.d. = 3.46		
subject × grammaticality type of learning phase:				subject × grammaticality type of learning phase:		
variance = 38.73, s.d. = 6.22				variance = 44.14, s.d. = 6.64		
residuals: variance = 24858.38, s.d. = 157.66				residuals: variance = 23690.45, s.d. = 153.92		

***p < 0.001.

**p < 0.01.

*p < 0.05.

### 3.3. What is the order of difficulty of learning?

The order of difficulty of the three item types was consistent with prediction: adjacent deterministic were easiest, then adjacent probabilistic, then non-adjacent deterministic. The confidence intervals overlapped for adjacent deterministic and adjacent probabilistic items, and for adjacent probabilistic and non-adjacent deterministic items, but did not overlap for adjacent deterministic and non-adjacent deterministic.

### 3.4. Are effects of adjacency and probability reflected in performance on the offline task?

The analysis was done on all the 42 participants' recall scores after the second session. Thirty-two participants showed uninformative patterns [i.e. 111 (all conditions correct) *n* = 16 or 000 (no conditions correct) *n *= 16]. One each showed a pattern that was inconsistent with the predicted order of difficulty (i.e. 101, 011 and 001). Seven showed a pattern consistent with the predicted order (i.e. 100, *n* = 5 and 110, *n* = 2). A *χ*^2^ test with one degree of freedom on the 10 cases with informative patterns showed that the proportion of participants who showed the predicted order of sensitivity on an offline task were significantly higher than 0.33 (the proportion expected if all six patterns are equally likely to occur), *χ*^2^ (1,42)= 6.19, *p* = 0.01. This shows that the order of difficulty is reflected on the recall task as well.

### 3.5. Does variation in sequential learning reflect stable individual differences?

**Figure 3.**
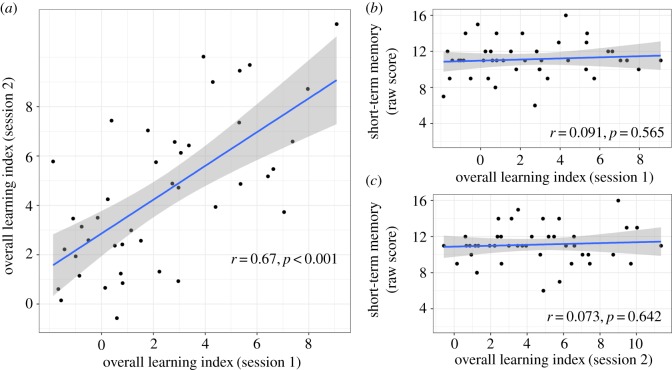
Correlation between (*a*) overall learning indices between sessions, (*b*) PSTM and learning indices on session 1, (*c*) PSTM and learning indices on session 2.

For each participant and each testing session, we calculated an overall index of learning. As can be seen in the individual plots (see https://osf.io/j35xt/), there was wide variation from person to person, with some showing no learning, some showing variable learning across conditions and others learning all dependency types. We used a regression discontinuity approach that combined data from all three grammatical conditions for each individual. This compares the slope of the learning phase with that of the break phase and gives a *t*-value that indicates how different the two slopes are. A high *t*-value indicates that the two phases were different, and learning occurred. The overall learning indices for the two sessions were significantly correlated, *r* = 0.67 (95% CI = 0.46 to 0.81), *n* = 42, *p* < 0.001 (figure 3*a* and table 2). The correlation coefficient exceeded the value of 0.65, which we had pre-specified as the cut-off for indicating acceptable test–retest reliability.

**Table 2.** Correlation matrix of learning conditions across sessions and with PSTM. With *N* = 42, *r* = 0.31 is significant at 0.05 level, *r* = 0.40 is significant at 0.01 level and *r* = 0.49 is significant at 0.001 level. In bold + italics: pre-registered confirmatory correlation. In bold: pre-registered exploratory correlations. Adj_D, adjacent deterministic; Adj_P, adjacent probabilistic; Non_D, non-adjacent deterministic; overall, overall learning index of Adj_D, Adj_P and Non_D.

	learning (session 1)				learning (session 2)				
	Adj_D	Adj_P	Non_D	overall	Adj_D	Adj_P	Non_D	overall	PSTM
learning (session 1)									
Adj_D	1.00	0.50	0.42	0.82	0.50	0.41	0.49	0.56	**0**.**18**
Adj_P		1.00	0.25	0.74	0.52	0.64	0.54	0.69	**0**.**11**
Non_D			1.00	0.72	0.31	0.15	0.36	0.30	−**0**.**04**
overall				1.00	0.59	0.53	0.59	***0****.****67***	**0**.**09**
learning (session 2)									
Adj_D					1.00	0.74	0.40	0.80	**0**.**09**
Adj_P						1.00	0.50	0.89	**0**.**01**
Non_D							1.00	0.77	**0**.**10**
overall								1.00	**0**.**07**

#### 3.5.1. Further analyses: 1. Registered-exploratory

##### 3.5.1.1. Is statistical learning capacity affected by STM?

We had predicted that phonological STM (PSTM) might relate to individual differences in learning, but the anticipated pattern and strength of correlation was uncertain. Phonological STM (raw scores from forward digit recall) did not correlate with any of the learning conditions individually or to the overall learning indices with either of the sessions (figure 3*b*,*c* and table 2).

#### 3.5.2. Further analyses: 2. Unregistered exploratory

##### 3.5.2.1. Learning in session 2

In our pre-registered analysis plan, we did not explicitly consider the session 2 data, which lend themselves to the same kind of analysis as session 1. Data on session 2 also showed grammaticality effect with all three grammatical conditions learned significantly compared to the reference condition. The order of difficulty was slightly altered compared to session 1 where non-adjacent deterministic was learned better than adjacent probabilistic in session 2. The confidence intervals overlapped for adjacent probabilistic and for non-adjacent deterministic conditions, but did not overlap for adjacent deterministic and non-adjacent deterministic conditions (table 2, model output for session 2).

##### 3.5.2.2. Speed of performance on offline task

We explored the speed of selecting (RT in seconds) the third item for accurate responses and explored if the proposed order of difficulty that was seen on online and recall accuracy could also be observed on the speed of recall. It must be noted that participants were told to take as much time as they want before selecting and asked to focus on being accurate. RT scores showed participants responded faster for the triplets that had one potential target (adjacent deterministic, median = 1.65, min = 0.80, max = 5.38, non-adjacent deterministic, median = 1.52, min = 0.98, max = 4.51) compared to that of triplets that had two potential targets (adjacent probabilistic, median = 2.28, min = 0.77, max = 5.64).

##### 3.5.2.3. Relationship between online and offline learning

As per the suggestions from reviewer 3, we explored the validity of the online measure of statistical learning by correlating with its explicit recall. This was done only for session 2, as explicit recall was not run after session 1. Correlation between overall learning index of session 2 with that of the sum of recall scores of each participant (note it is discrete and can vary from 0, 1, 2, 3) showed a high correlation between these, *r* = 0.80. Since one of the variables was continuous and the other was discrete, we converted both the variables into discrete using *discretize* function in R and ran the correlation.

##### 3.5.2.4. Internal consistency of online measure

As per the suggestions from reviewer 2, we explored the internal consistency of our task by a split-half procedure. We used the same regression discontinuity procedure to extract the *t*-values showing the difference in slope across two phases but separately for odd and even sets for each grammatical condition. This showed a high correlation between odd and even trials for all three grammatical conditions (adjacent deterministic, *r* = 0.80; adjacent probabilistic, *r* = 0.65; non-adjacent deterministic, *r* = 0.75).

All the participants reported that they noticed the patterns and the broken sequence. However, note that only 26 participants (61%) could recall the third item of at least one of the four test triplets accurately. Some participants wanted to talk about the pattern they observed after the first session, which the examiner tried to not encourage. This may be crucial in suggesting that some participants might have been looking for patterns right from the beginning on session 2 when the same task was given.

## 4. Discussion

### 4.1. Registered

In the present study, we presented auditory triplets of familiar words in three types of grammatical sequences [adjacent deterministic (control), adjacent probabilistic and non-adjacent deterministic], keeping the frequency of the predictor items constant across triplets in a within-subject design. We also had random (baseline/ungrammatical) triplets. We predicted that a grammaticality effect would be seen, and within the grammatical conditions, adjacent deterministic would be the easiest to learn followed by adjacent probabilistic and non-adjacent deterministic conditions. We also tested the participants again (after at least 3 days) on the same task with different triplets and predicted that our task would show reliable (i.e. *r* ≥ 0.65) individual differences in online statistical learning. Finally, we proposed to explore the relation between phonological short-term memory (PSTM) and statistical learning.

Our results confirmed there is a grammaticality effect, i.e. individuals are capable of incidentally extracting regularities from an audiovisual stream when the stream includes statistical structures of different complexities. The effect has been well documented using offline (e.g. [14,39]) as well as online approaches [13,26,32]. The effect was proposed as a minimal requirement to show that our task is able to detect individuals' sensitivity to regular compared to irregular properties in the input. In contrast with prior methods, such as Vuong *et al.* [13], our approach allows us to document the course of learning, showing differential speeding up between grammatical conditions as a function of statistical complexity and exposure. Note Vuong *et al*. had looked at the overall RT difference across blocks between two types of structures—adjacent probabilistic and non-adjacent probabilistic—and had predicted that adjacent structures would be easier to learn compared to non-adjacent structures. Their prediction was supported by offline but not by online scores. We found better learning of adjacent than non-adjacent sequences in both online and offline measures. The failure to see online effects in the Vuong *et al*. study may be attributed to the fact that their measure was not sensitive to the course of learning. An approach that takes into account the extent of learning (see also [32]) of each condition (over blocks) compared to the baseline condition suggests that an online task can capture the predicted order of difficulty (i.e. adjacent deterministic < adjacent probabilistic < non-adjacent deterministic).

Our findings of order of difficulty on an online task, in part, could be compared to Siegelman *et al*.'s [32] (experiment 2). In one experiment, these authors presented 12 visual (abstract shape) pairs (predictor–predicted item) that contained three adjacent TP conditions (TPs of 1, 0.8 and 0.6). They equalized the number of repetitions across TP conditions and their intentional familiarization stream consisted of 30 blocks, with all 12 pairs appearing once in each block. On their self-paced familiarization phase, participants actively pressed spacebar to propel to the next shape. They also were administered an offline 2AFC task and documented the developed sensitivity to these TPs post-familiarization. They found that the RTs for the predicted items were faster than predictor items and within the predicted items, the order of difficulty between TPs was 1 < 0.8 < 0.6. Note, however, they were not able to demonstrate this differential sensitivity on the 2AFC task.

Our findings on test–retest reliability indicate that our task is reliable (*r* = 0.67) and that the learning documented in this approach is a fairly stable signature of an individual. Learning of specific dependency types is also reasonably reliable across sessions (table 2). The internal consistency of the present task is better (0.65 and above) compared to the ones reported in West *et al*. [19] who reported poor internal consistency on some of the implicit learning tasks. To our knowledge, Siegelman *et al.* ([32], experiment 1a and b), who found a correlation of 0.64 between two testing sessions, is the only experiment that looked at the test–retest reliability of an online SL approach. However, note that our study differs from Siegelman *et al*. in two respects. First, learning in Siegelman *et al*. could be considered ‘intentional’, whereas our participants were not told about the patterns. Second, Siegelman and colleagues in their experiment 1a and b for test–retest reliability had only one dependency structure, that is, the TPs within triplets were always 1 and between triplets were 0, whereas in the present study, we included a range of dependencies.

The present study did not find a relation between PSTM and SL. Note we did not have specific predictions for the relationship and stated this correlation as exploratory. The findings are largely in line with the literature that suggests that general memory scores do not correlate with incidental sequence learning [40–42]. Our present findings may be used to argue for the view that SL, at least when tapped incidentally, could be a unique facet of an individual's ability which may be independent of one's memory abilities [43].

### 4.2. General points

We also measured the validity of our online task by correlating the overall online learning index (of session 2) with offline scores (after session 2) which showed a high correlation, suggesting that our online approach is psychometrically valid [32]. Within the recall, the third item of the adjacent deterministic triplet was recalled by 24 people compared to adjacent probabilistic and non-adjacent deterministic (19 each) triplets, suggesting that more people developed conscious knowledge for adjacent deterministic condition. The higher RT for recalling adjacent probabilistic (where there were two potential targets) may suggest that participants were indeed aware that certain triplets led to more targets than others—a conscious aspect. However, caution must be taken in interpreting the degree of consciousness involved in the recall task. Note that we did not ask the participants to recall all three items of triplets, which is the norm in implicit literature (e.g. [44]). Rather, during recall, our participants were prompted to select the third item from a range of distractors given the first two words. As argued in the introduction, this kind of procedure was adapted to reduce the load on memory, keeping in mind, future experiments on children. The processes tapped resemble those involved in the serial pattern production paradigm [30], where if there was any memory trace developed for a sequence, the activated nodes for the first two items of the triplet might trigger response to the third item.

Now, we explore the findings on order of difficulty between two sessions and relate it to two prevalent views of non-adjacency learning in statistical learning, Gómez's [12] and Romberg & Saffran's [14]. Note that the participants who showed learning in session 1 might have been looking for patterns (note after the first session some participants wanted to talk about the patterns) right from the beginning on session 2. Findings show that on session 1, adjacent probabilistic was learned better than non-adjacent deterministic condition (the predicted order of difficulty). However, on session 2, non-adjacent deterministic was learned better than adjacent probabilistic (table 1 for coefficients, *t*-values for session 2). On both the occasions, adjacent deterministic was the best learned condition. Our findings on session 1 could be argued to support Gómez's [12] argument that individuals will develop sensitivity to non-adjacent dependencies only after developing sensitivity to adjacent dependencies. That is, they keep searching for the non-variable items to form dependencies. Findings on session 2 also support the view of Romberg & Saffran [14] who argued that the learning for adjacent and non-adjacent distributions emerge as a different distribution. That is, first they extract what is consistent irrespective of distance. The only difference that might have contributed to this discrepancy is awareness to the patterns. This aligns with the differences in instructions on Gómez's and Romberg's study, where the earlier was incidental (the exposure phase) and the later was intentional (but see [8]). On both the sessions, adjacent conditions correlate moderate to highly (table 2) with non-adjacent condition, suggesting that the present data are more in favour of Gómez's continuous distribution argument (but see Siegelman *et al.* [32]).

To our knowledge, this is the first online incidental SL task that uses familiar, meaningful items. We adopted this approach because the present study is part of a research programme that examines statistical learning in children and ties their SL abilities to their language abilities. One of the objectives was to develop an SL task that could be motivating and fun for children and where learning focused just on sequences without requiring any processing of unfamiliar materials. Further, where test–retest reliability of other SL tasks for children has been examined, it has been found to be low (see [19]), so we deemed it important to establish the test–retest reliability of our novel SL task. Our goals were largely achieved. The task is successful in showing that an individual's sensitivity to different statistical properties is trackable, and individual differences in sensitivity are reasonably stable. However, it is worth noting that the present design needs improvement to inform finer details regarding the learning dynamics such as the time point at which the learning of a particular grammatical condition was learned relative to a reference (ungrammatical) condition, and whether one condition shows greater learning speed compared to another. This is important for future research in children with developmental language disorders as one of the questions that remains to be answered in this population is what type of statistical properties are particularly harder for children and what are their specific strengths and weaknesses. In order to study such a specific question, the present study has made a good start.

## 5. Pilot data

We initially gathered pilot data (adults, *n* = 10) on an earlier version of the task. This found an overall effect of grammaticality, but the data were noisy and we made more modifications to the task as a consequence, to minimize item-specific effects (e.g. ensuring that pictures in each array had names that started with a different phoneme and devising different presentation orders so that items could be counterbalanced across participants and sessions. We subsequently piloted a range of versions of the task, to take into account reviewer suggestions, leading us to settle on the current version of the task, which included three sequence types, as well as random sequences, in a format that required a response to just the final item in a triplet sequence. Pilot data for this version of the task were obtained for nine participants, and confirmed that learning was clearly observed for a subset of participants in some conditions, but others showed little indication of learning (see osf.io/342rz).

The approved IPA manuscript after stage 1 review can be found at https://osf.io/ht8gq/.

## Data accessibility

The Matlab code that runs the learning task can be accessed at https://osf.io/u892g/. Excel files showing words used per each trial across three routines/sessions, materials related to raw pilot data, preliminary R script for power, data and statistical analysis can be accessed at https://osf.io/j6syy/. Final raw data .csv files, supplementary graphs showing individual learning plots across sessions, and updated analysis script (including offline scores analysis) can be accessed at https://osf.io/v2kws/.
